# A Data Preparation Methodology in Data Mining Applied to Mortality Population Databases

**DOI:** 10.1007/s10916-015-0312-5

**Published:** 2015-09-18

**Authors:** Joaquín Pérez, Emmanuel Iturbide, Víctor Olivares, Miguel Hidalgo, Alicia Martínez, Nelva Almanza

**Affiliations:** Tecnológico Nacional de México / CENIDET, Interior Internado Palmira s/n, Palmira, 62490 Cuernavaca, Morelos Mexico; Universidad Politécnica de Madrid, ETSII, Boadilla del Monte, Madrid, Spain

**Keywords:** Data preparation methodology, Mortality databases, Censuses databases, Epidemiological data mining

## Abstract

It is known that the data preparation phase is the most time consuming in the data mining process, using up to 50 % or up to 70 % of the total project time. Currently, data mining methodologies are of general purpose and one of their limitations is that they do not provide a guide about what particular task to develop in a specific domain. This paper shows a new data preparation methodology oriented to the epidemiological domain in which we have identified two sets of tasks: General Data Preparation and Specific Data Preparation. For both sets, the Cross-Industry Standard Process for Data Mining (CRISP-DM) is adopted as a guideline. The main contribution of our methodology is fourteen specialized tasks concerning such domain. To validate the proposed methodology, we developed a data mining system and the entire process was applied to real mortality databases. The results were encouraging because it was observed that the use of the methodology reduced some of the time consuming tasks and the data mining system showed findings of unknown and potentially useful patterns for the public health services in Mexico.

## Introduction

Data mining has demonstrated to be an activity of interest because it allows the exploration of high volumes of data in order to extract implicit, potentially useful and previously unknown information [1]. However, the quality of the hidden knowledge depends on correct data preparation.

Authors pointed out that data preparation takes about 50–70 % [2], sometimes 80 % [3] or even up to 90 % of the time, because real data may be incomplete, noisy and inconsistent [4]. Therefore, the experts require systematic and detailed methodologies and automated tools in order to reduce the effort required to perform this phase.

Related works on data preparation methodologies are limited. In [2], the authors proposed a process model that can be used in several domains; however, its level of detail is very general for each phase and sub-phase. The sub-phases of the data preparation do not describe the particular tasks for a given domain nor the order of how to execute them. Other methodologies [3, 5, 6] are focused on a particular data preparation sub-phase, data cleaning or data selection.

In [7], the authors use population and epidemiological cancer databases in order to establish and display, cartographically, possible relations between patients with cancer and their proximity to factories and cell phone antennas.

In the healthcare domain, there is research [8–10] that uses epidemiological databases and shows the uses of them in data mining projects. In [11], the authors proposed a data preparation method that uses clustering and classification techniques for eliminating inconsistent data. Their methodology was applied on heart and diabetes datasets.

In [12], the authors suggest the development of new data mining methodologies for the social sciences domain due to the limitations of current methodologies. The authors in [13] describe an application that uses epidemiological data to identify patterns of regions with a high incidence rate of five diseases, defined by local medical associations. Similar to our research, the application visually shows the model result.

Our paper proposes a data preparation methodology consisting of a general and a specific part. The general part could be reused in other domains; however, the specific part is oriented to the epidemiological domain. Additionally, a data preparation tool that automates some tasks for the epidemiological domain is described. A practical case study, using mortality population databases, was developed with the purpose of discovering clusters of districts with high mortality rates of cancer in Mexico.

## Data preparation methodology for epidemiological data mining

The sections below show: a) the description of the proposed methodology, and b) a practical illustrative example study using mortality population databases and a data preparation subsystem.

### Description of the methodology

**Fig. 1:**
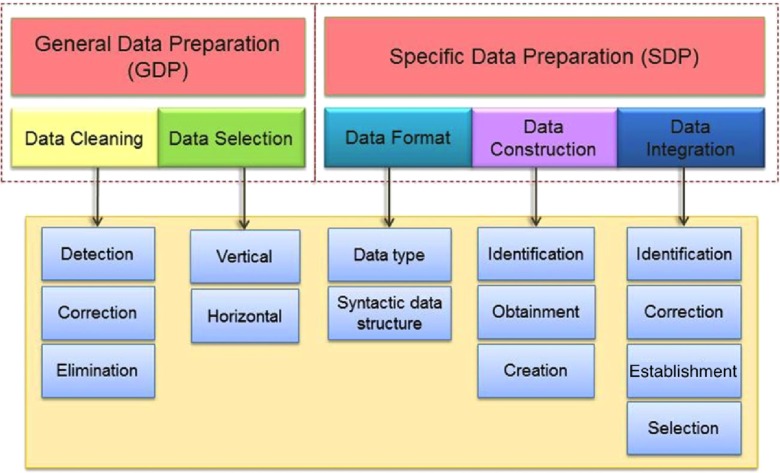
Tasks of the data preparation methodology

This methodology is derived from the analysis made of the related work (particularly CRISP-DM was followed as a guideline). We defined particular tasks for each data preparation sub-phase and proposed to separate the data preparation sub-phases into two sets of tasks to be performed. The description of Fig. 1 is explained as follows.

**Fig. 2:**
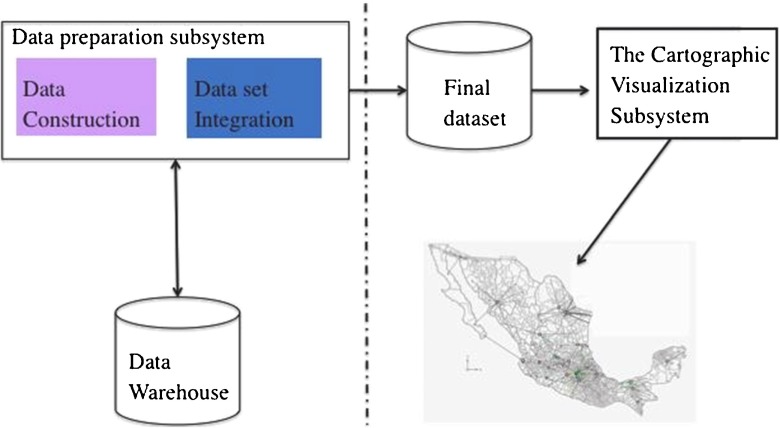
Conceptual diagram of the data preparation subsystem

#### General Data Preparation (GDP)

GDP refers to a set of data preparation tasks that are independent from the specific data mining goal to be achieved. This set tries to uniform and select, from several sources, the data that will be used. The tasks included are:

1. Data cleaning. The principal tasks to develop are: detection, correction and elimination of anomalies or outliers in the values of records and attributes.
2. Data selection. This includes vertical (attributes) and horizontal (records) selection [14].

This set of tasks could be applied in other domains because of their generality.

#### Specific Data Preparation (SDP)

SDP particularizes a set of tasks which have a closer relation with the data mining goal to be achieved in this research. This set comprises the tasks:

1. Data format. Changing the data type and the syntactic data structure of the attributes and values (if required).
2. Data construction. Identifying, obtaining and creating new attributes (if needed) or filling out missing values through arithmetic operations.
3. Data integration. Identifying, correcting of integration conflicts, establishing data relations and selecting the data integration schema [15].

These tasks are oriented to the epidemiological domain and could be used in other data mining applications for this domain.

### Methodology validation

Data mining of healthcare data is one of the most rewarding and challenging areas of application in data mining and knowledge discovery [8]. Epidemiological databases are large, complex, irregular time series and vary in quality. As a practical case study, official mortality databases from 2000 in Mexico were utilized. We developed the data preparation process, initially in a manual way and then, we chose some tasks in order to be automated as shown in the sections below.

#### Manual data preparation

The databases are briefly described in Table 1. We performed either the GDP or the SDP or both tasks for all the databases. The process for the GDP was manually developed, and it is briefly described below:

1. Data cleaning. For each data file, we identified and deleted headlines, which did not provide information of interest. In population and geographical databases, we identified that some records were displaced from their respective attributes which were then corrected and assigned to their respective attributes.
2. Data selection. 31 out of 38 attributes were removed from the mortality database because they did not provide information of interest according to experts’ domain (vertical selection). Although, we deleted all the records related to districts with populations less than 100,000 inhabitants (horizontal selection).

**Table 1:** Description of the population-based databases

Database	Attributes	Records	Description
Mortality	38	437,667	Deaths occurring
			in 2000.
			SINAIS-INEGI [16].
Geographic	7	2,475	Geographical
			position of the
			districts of Mexico.
			SIMBAD-
			INEGI [17].
Population	3	2,475	Total population by
			district in Mexico.
			INEGI [18].
ICD	24	2,049	International
			Classification
			of Diseases.
			CEMECE [19].

These tasks were not automated because of the number of decisions that the data miner and the domain experts had to make.

For SDP, the tasks developed were:

1. Data format. For the attributes Latitude and Longitude, we changed the data type from sexagesimal degrees to a decimal representation (for research purposes).
2. Data construction. These tasks were automatically performed using the data preparation subsystem described in the *Automatic data preparation* section. The subsystem performed the following operations:
  Calculation of the mortality incidence. Obtained by counting the number of deaths per district for a specific cause of death (*Incidence*).
  Calculation of the mortality rate. Equation 1 was used for calculating it (*Rate*).
    1 $$ Rate = \frac{Incidence}{Population} * 100,000 $$
  Where *Population* is the total inhabitants per district.
3. Data integration: We developed two tasks:
  Data warehouse integration. We integrated all the databases (see Table 1) in a data warehouse. We chose a star schema and created a fact table with the following attributes: Key (district key), Cause (cause of deaths), Year (when those deaths occurred), Incidence (number of deaths per district) and Rate (mortality rate per district). Previously, we established data relations through the common attribute Key (district key).
  Final dataset integration. It is a file that contains the information for a specific cause of death (Cause), the mortality rate (Rate) and the geographical position (Latitude and Longitude) for all the districts, with a population greater than 100,000.

From this second set of tasks, we chose data construction and data integration to be automated. The calculations and operations to be performed represent the largest effort because they are the most time consuming tasks. In addition, they must be repeated for each cause of death.

#### Automatic data preparation

We implemented a data preparation subsystem in the Java programming language using the Java Database Connectivity and the Structured Query Language to establish the connection between the application and the data warehouse. The subsystem architecture is composed by:

1. Data construction module. It selects all the records related to a specific cause of death and executes the calculation of mortality incidence and the calculation of mortality rate. The results are permanently stored in the fact table.
2. Data integration module. It selects all the records for the given cause of death and then creates a final dataset which is used as input by the cartographic visualization subsystem (described in [20]).

Figure 2 shows the conceptual diagram of the data preparation subsystem and its interaction with the cartographic visualization subsystem. The cartographic visualization subsystem converts the final dataset and applies to it the K-means algorithm, which partitions the dataset into clusters of districts with similar Latitude, Longitude and Rate; and finally, it draws on a map all the final clusters to determine the cause-space mortality.

## Results and contributions

### Results analysis

As a result of the manual data preparation process performed, we implemented a data warehouse with information of all the deaths that occurred in 2000 in Mexico, with a total of 2,049 different causes, in districts with a population of over 100,000 inhabitants.

We selected one cause of death (C16, stomach cancer) and the results obtained were compared with results of previous works [20]. The values generated, in an automatic way, for *Incidence* and *Rate* by the data preparation subsystem match exactly, the values obtained manually, in previous works.

For the cause of death C16, two clusters of interest with the highest mortality rates are were identified. Table 2 shows a cluster in the northwest region of Mexico between the states of Sonora and Baja California Sur. Table 3 shows another cluster which is located between the states of Chiapas, Tabasco and Veracruz. The specialized literature reported high mortality rates in this zone, mainly in the Chiapas highlands in the southeast region of Mexico, caused by the bacterium Helicobacter Pylori [21].

**Table 2:** Cluster of interest for the cause of death C16

Name of the District	Incidence	Rate
Guaymas	15	11.52
Hermosillo	48	7.87
La Paz	14	7.11
Los Cabos	7	6.64

**Table 3:** Cluster of interest for the cause of death C16

Name of the District	Incidence	Rate
Minatitlan	14	9.15
Comalcalco	14	8.50
Tapachula	21	7.73
San Cristobal	9	6.80
Macuspana	9	6.72
Tuxtla Gutierrez	28	6.45

**Fig. 3:**
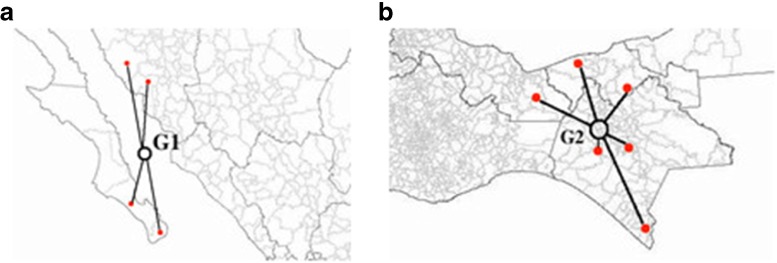
Clusters of interest for the cause of death C16

The red points in Fig. 3, northwest region-a, (Table 2) and southeast region-b, (Table 3) represent the districts that belong to each cluster, denoted by the centrally-located black circles G1 and G2.

Table 4 shows an example of the dataset used by the K-means algorithm. The values correspond to the Latitude, Longitude and Rate attributes.

**Table 4:** Dataset example

Latitude	Longitude	Rate
19.39073	−99.14361	7.0031
18.92133	−99.23468	4.6957
19.03247	−98.19576	2.6367
...	...	...

Additionally, another inherent contribution of this research was the automation of three tasks of the Specific Data Preparation set (SDP). These tasks were performed manually; then, they were performed automatically using the data preparation subsystem and the elapsed time was registered. Table 5 shows the time comparison of the tasks when being executed manually and automatically.

**Table 5:** Time comparison for the data preparation tasks for cause C16

Task	Manual	Automated	Reduction
	(min)	(min)	(%)
Mortality			
Incidence	33.53	0.058	99.83
Mortality			
Rate	5.16	0.330	99.61

Columns 2 and 3 show the time taken in minutes by the manual and automated calculations. The results show an important reduction of the time due to the methodology that helps us to identify which tasks were susceptible to automate.

### Generality of the methodology

In this research, we used official and international standards. Therefore, we consider that this methodology can be applied in other countries meeting the following requirements:

- The mortality data comply with ICD.
- Select populations with more than 100,000 inhabitants for epidemiological studies purposes.
- The cartographic data of those populations are expressed according to official coordinate systems.

## Conclusion and future work

This paper shows that it is feasible to develop a data preparation methodology oriented to the epidemiological domain. The most important contribution is the identification of fourteen specialized tasks and two sets of tasks in the data preparation phase. The identification of GDP and SPD helps data mining experts to reuse tasks for the epidemiological domain (where a population data is used).

Based on the promising results, we contrasted the quality of the findings with those reported by experts from the epidemiological domain. Consequently, these results could help them in decision-making processes at the time of conducting primary care campaigns, especially oriented to those districts in Mexico with high mortality rates. Health services could have a new perspective of how these diseases affect some particular regions.

Finally, we consider that this methodology may be applicable and useful in other countries since the appropriateness of the methodology referred to in this work could be adjusted by relatively simple changes.
